# Coelomocytes of the Oligochaeta earthworm *Lumbricus terrestris* (Linnaeus, 1758) as evolutionary key of defense: a morphological study

**DOI:** 10.1186/s40851-023-00203-y

**Published:** 2023-03-04

**Authors:** Alessio Alesci, Gioele Capillo, Angelo Fumia, Marco Albano, Emmanuele Messina, Nunziacarla Spanò, Simona Pergolizzi, Eugenia Rita Lauriano

**Affiliations:** 1 Department of Chemical, Biological, Pharmaceutical and Environmental Sciences, University of Messina, 98166 Messina, Italy; 2 Department of Veterinary Sciences, University of Messina, 98168 Messina, Italy; 3 Institute of Marine Biological Resources and Biotechnology, National Research Council (IRBIM, CNR), 98164 Messina, Italy; 4 Department of Clinical and Experimental Medicine, University of Messina, Padiglione C, A. O. U. Policlinico “G. Martino”, 98124 Messina, Italy; 5 Department of Biomedical, Dental and Morphological and Functional Imaging, University of Messina, 98125 Messina, Italy

**Keywords:** Annelid, Coelomocytes, Earthworm, Evolution, Immunity

## Abstract

Metazoans have several mechanisms of internal defense for their survival. The internal defense system evolved alongside the organisms. Annelidae have circulating coelomocytes that perform functions comparable to the phagocytic immune cells of vertebrates. Several studies have shown that these cells are involved in phagocytosis, opsonization, and pathogen recognition processes. Like vertebrate macrophages, these circulating cells that permeate organs from the coelomic cavity capture or encapsulate pathogens, reactive oxygen species (ROS), and nitric oxide (NO). Furthermore, they produce a range of bioactive proteins involved in immune response and perform detoxification functions through their lysosomal system. Coelomocytes can also participate in lithic reactions against target cells and the release of antimicrobial peptides. Our study immunohistochemically identify coelomocytes of *Lumbricus terrestris* scattered in the epidermal and the connective layer below, both in the longitudinal and in the smooth muscle layer, immunoreactive for TLR2, CD14 and α-Tubulin for the first time. TLR2 and CD14 are not fully colocalized with each other, suggesting that these coelomocytes may belong to two distinct families. The expression of these immune molecules on Annelidae coelomocytes confirms their crucial role in the internal defense system of these Oligochaeta protostomes, suggesting a phylogenetic conservation of these receptors. These data could provide further insights into the understanding of the internal defense system of the Annelida and of the complex mechanisms of the immune system in vertebrates.

## Introduction

The internal defense mechanisms allow the survival of the host in the metazoan. These mechanisms become increasingly complex as organisms evolve. Unlike vertebrate deuterostomes, protostomes lack adaptive immunity, basing their defense on innate immunity [56]. The initial line of defense against bacteria and microorganisms in vertebrates is the quick activation of innate immune cells and molecules. Recently, invertebrates have become candidates for innate immunity analysis to reveal strategies and complexities of vertebrate adaptive immune response. Invertebrates have developed a variety of active immune mechanisms including the production of antimicrobial peptides, coagulation, phagocytosis, and encapsulation reactions [20, 24]. These mechanisms depend on “pattern recognition receptors” (PRRs), innate immune molecules which can discriminate “self” from “non-self” membrane components. The recognition of “pathogen-associated molecular patterns” (PAMPs) regulates the start of essential signaling pathways, up to the activation of genes encoding inflammatory mediators, antimicrobial peptides, and phagocytosis regulators [32]. Oligochaetes annelids (earthworms) have become a model for comparative immunology, and, like leeches, are used for terrestrial and aquatic environmental biomonitoring (B.-T. [48, 52, 53, 72]. Earthworms are provided with innate, highly developed, and effective defense mechanisms against dangerous environmental microorganisms. The first mechanical–biological line of defense, that separates the body of annelids from the external environment is the tegument, composed of an epidermis and muscle layers. It has a cuticle with a thin layer of mucopolysaccharides and proteins, which act as an antimicrobial barrier [20]. In earthworms the cellular and humoral immune systems work together for the first time in the metazoans evolution. The fluid in the coelomic cavity is thought to be responsible for humoral immunological activities. The coelomic cavity of earthworms is filled with fluids containing free and wandering coelomocytes and their pigmented aggregates, called brown bodies, product of the encapsulation of invading bacteria and particulate residues. Several biological processes, including hemolytic, proteolytic, cytotoxic, and antibacterial are performed by the coelomic fluid of earthworms [19, 24, 29]. The coelom directly communicates with the external environment through the dorsal pores and the coupled nephridial tubules, that excrete the metabolites. These pores are also involved in the elimination of bacteria and exhausted coelomocytes. Under stress conditions, the coelomic fluid and the suspended cells can be rapidly expelled by the increase in intracelomic pressure [24].

Coelomocytes of earthworms are an essential element of the cellular innate immunity. These cells resemble vertebrate leukocytes in both morphology and function [33]. Coelomocytes perform a wide range of tasks, including cell cytotoxicity, encapsulation, and phagocytosis. They can be divided into two primary subpopulations: eleocytes and amoebocytes (hyaline and granular) [33]. Hyaline and granular amoebocytes are involved in phagocytosis and encapsulation, expressing various PRRs [23, 60]. These circulating cells that migrate into tissues from the coelomic cavity are very similar to vertebrate macrophages, engulfing or encapsulating pathogens, reactive oxygen species (ROS), and nitric oxide (NO) [39]. Eleocytes produce a wide range of bioactive proteins that are involved in the humoral immune response and also detoxifying the body through their lysosomal system [24]. Coelomocytes can take part in lithic reactions against target cells and the release of antimicrobial peptides. Among the immune mechanisms, transplantation experiments have demonstrated the existence of self-recognition in earthworms [28].

Annelid coelomocytes rapidly form immune mechanisms against bacteria, parasites, and yeasts through cellular reactions [59]. This rapid response is mediated by soluble antibacterial molecules, such as lysozyme, lysenin, and lumbricin [22, 42, 68]. Highly conserved immunological molecules, such as cell surface indicators, were discovered on coelomocytes using cytometric flow analysis. In particular, coelomocytes reacted with mammalian specific antibodies, showing superficial positivity to antibodies anti-CD11a, CD45RA, CD45RO, CDw49b, CD54, b2-M, and Thy-1 [30, 34].

Our study aims to immunohistochemically evaluate the presence of TLR2, CD14, and α-Tubulin for the first time in coelomocytes of *Lumbricus terrestris* (Linnaeus, 1758), a broad, multi-segmented, cylindrical earthworm belonging to the family Lumbricidae (Annelida, Oligochaeta), hermaphrodite, 8 to 10 cm long [55]. Improving the knowledge of the invertebrate internal system of defense can help in the understanding of the more sophisticated immunity of vertebrates and, consequently, the evolution of the immune response [63].

## Materials and methods

### Samples and Tissue preparation

*Lombricus terrestris* samples come from our laboratory histotheca. Previously, they were taken in uncontaminated open field and acclimated for two weeks in fresh soil, as reported by Licata et al. [51], and then were prepared using the standard methods for light microscopy. Samples were immersed immunofix (paraformaldehyde 4%) (05-W01030705, BioOptica Milano S.p.A., Milan, Italy) for two hours. Then, they were treated with a dehydrating ethanol scale (from 30 to 100% ethanol), and then prepared for the inclusion in paraffin using xylene. Once included, two thin sections of 3–5 μm cutted with a rotary microtome were placed on each slide.

### Histology

A morphological and a histochemical stains were used to process the slides [4, 6]. Slices were then rehydrated using progressive alcohol solutions (from 100 to 30% ethanol), to distilled water after being deparaffinized in xylene [17],G. [75], subsequently, they were stained using Mallory trichrome and AB/PAS [3]. After staining slides were mounted using Eukitt (BioOptica Milano S.p.A, Milan, Italy, Europe). Data on the stains used in this study are included in Table 1.

**Table 1:** Stains used in this study

Stain name	Type	Catalogue number	Supplier
*Mallory trichrome*	Morphological	04–020,802	BioOptica Milano S.p.A, Milan, Italy, Europe
*Alcian blue/Periodic Acid Schiff (AB/PAS)*	Histochemical	04–163,802	BioOptica Milano S.p.A, Milan, Italy, Europe

### Immunofluorescence

Deparaffinized sections were treated with a 5% solution of sodium borohydride to eliminate autofluorescence, then a 2.5% solution of bovine serum albumin (BSA) was used, and then slices were incubated overnight with primary antibodies against TLR2, CD14, and α-Tubulin [40]. After that, the incubation of secondary antibodies was performed. To avoid bleaching, the slices were mounted using Fluoromount™ Aqueous Mounting Medium (Sigma-Aldrich, Taufkirchen Germany, Europe). Experiments were run without the primary antibodies as a negative control (data not shown). In order to confirm that the primary antibodies were immunopositive, rat skin tissues were employed as a positive control (data not shown) [5, 47, 74].

The sections were analyzed under a Zeiss LSM DUO confocal laser scanning microscope with a META module (Carl Zeiss MicroImaging GmbH, Jena, Germany, Europe) with a helium–neon (543 nm) and argon (458 nm) lasers of different wavelengths [7]. To improve pictures Zen 2011 (LSM 700 Zeiss software, Oberkochen, Germany, Europe) was used. Using Adobe Photoshop CC version 2019 (Adobe Systems, San Jose, CA, USA) the digital images were merged to a composite figure. The fluorescence intensity curves were then evaluated using Zen 2011 "Display profile" feature [58]. Details about antibodies are summarized in Table 2.

**Table 2:** Antibodies data

Antibody	Supplier	Dilution	Animal source
CD14	Santa Cruz Biotechnology, Inc., Dallas, TX, USA	1:200	Mouse
TLR2	Active Motif, La Hulpe, Belgium, Europe	1:125	Rabbit
α-Tubulin	Santa Cruz Biotechnology, Inc., Dallas, TX, USA	1:500	Mouse
Alexa Fluor 488 Donkey anti-Mouse IgG FITC conjugated	Molecular Probes, Invitrogen	1:300	Donkey
Alexa Fluor 594 Donkey anti-Rabbit IgG TRITC conjugated	Molecular Probes, Invitrogen	1:300	Donkey

### Quantitative Analysis

Data were gathered for the quantitative analysis through the examination of ten sections and twenty fields per sample. The cell positivity and number was assessed using ImageJ software 1.53e. The number of coelomocytes positive for TLR2, CD14, and α-Tubulin was counted using SigmaPlot version 14.0 (Systat Software, San Jose, CA, USA). The normally distributed data were analyzed using one-way ANOVA and a Student's t-test. Mean values and standard deviations (SD) of the number of immunoreactive coelomocytes are reported: ** *p* ≤ 0.01, * *p* ≤ 0.05.

## Results

**Fig. 1:**
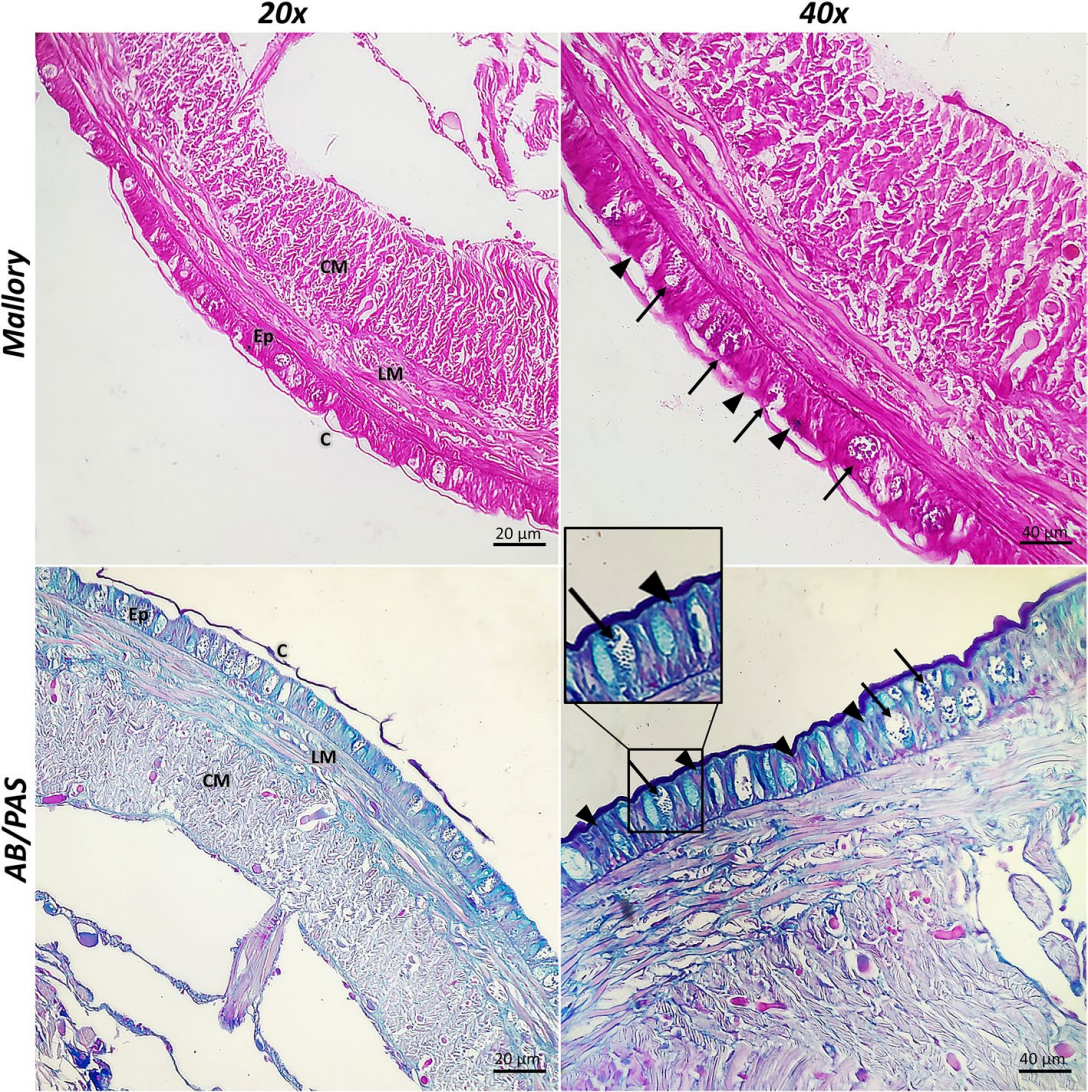
Cross Sects. (5 µm thick) of *Lumbricus terrestris*, 20x and 40x. These sections were stained with Mallory Trichrome and AB/PAS demonstrating the architecture of the cuticle (C), the epidermis (Ep), longitudinal muscle (LM), and circular muscle (CM). The epidermis is morphologically well-defined; it is either a single epithelial layer or pseudostratified; it consists of supporting epithelial cells (arrowheads) and mucous glandular cells (arrows)

The earthworm *Lumbricus terrestris* has a mono-stratified epithelium and a covering fibrous cuticle that constitute the epidermis. Connective and muscular tissues that compose most of the body wall lie beneath the epidermis. The epidermal epithelium keeps the collagenous cuticle distinct from the rest of the organism and is responsible for the production of this cuticular collagen. The cuticle, the epidermis, the longitudinal muscle, and the circular muscle are all visible by histological inspection of the cross-section of the body wall of earthworms. The epidermis is morphologically well-defined; it is either a single epithelial layer or pseudostratified; it consists of supporting, basal, glandular, and sensory cells (Fig. 1).

**Fig. 2:**
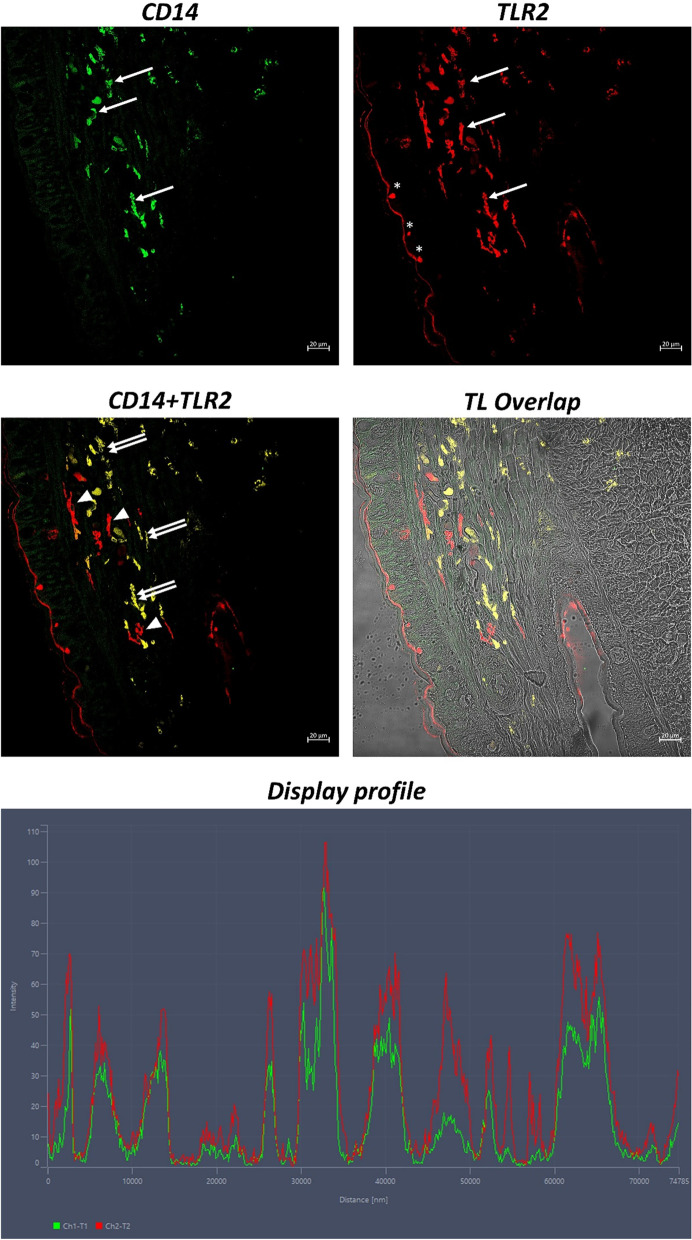
Cross Sects. (5 μm thick) of *Lumbricus terrestris*. Sections are immunohistochemically treated with anti-TLR2 and anti-CD14 antibodies. Magnification 20x. Clear coelomocytes immunoreactive for antibodies tested (arrows) are evident. Epithelial cells are labeled by TLR2 (*). Some coelomocytes are positive only for TLR2, maybe indicating a different cell population, probably eleocytes (arrowheads). Positive colocalization of these antibodies may suggest that these cells are amoebocytes (double arrows). The “Display profile” function confirms these data. TL = Transmitted Light

**Fig. 3:**
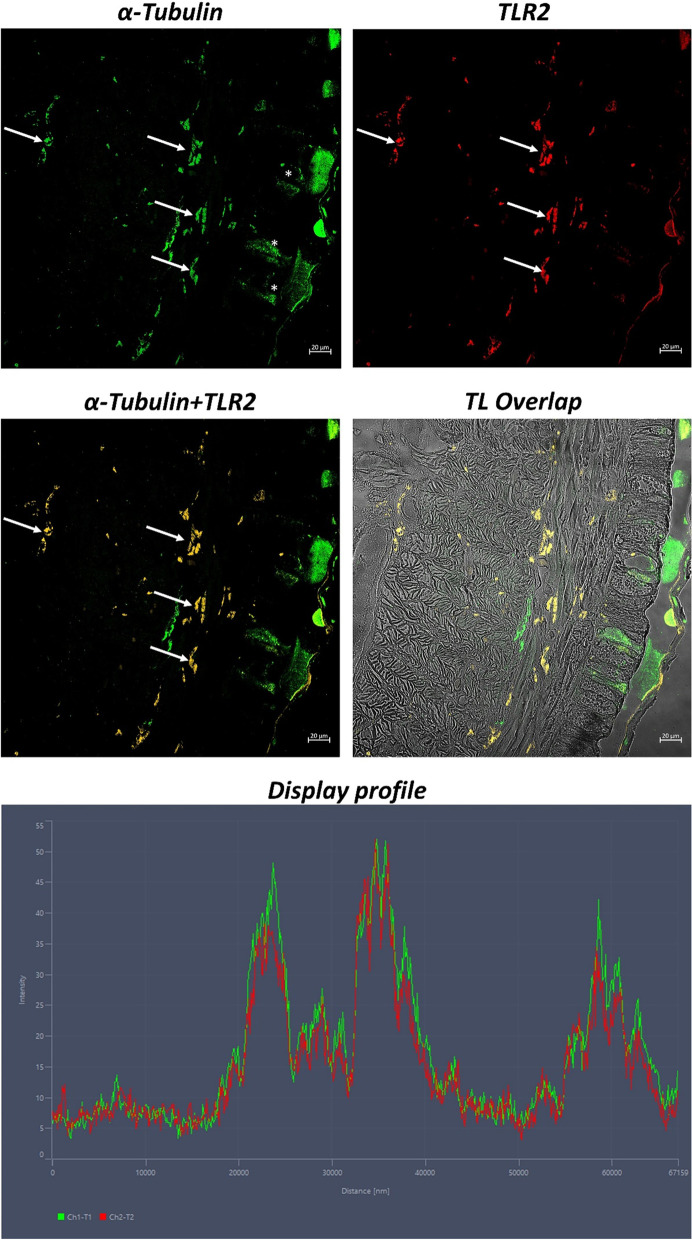
Cross Sects. (5 μm thick) of *Lumbricus terrestris*. Sections are immunohistochemically treated with anti-TLR2 and anti-α-Tubulin antibodies. Magnification 20x. Coelomocytes immunoreactive for antibodies tested are evident and colocalized (arrows). In addition, some glandular mucous cells are positive for α-Tubulin (*). The “Display profile” function confirms these data. TL = Transmitted Light

Immunofluorescence analysis, at the confocal microscope observation, show positive coelomocytes for TLR2 (Figs. 2, 3), CD14 (Fig. 2), and α-Tubulin (Fig. 3), scattered in the epidermal and the connective layer below, both in the longitudinal and in the smooth muscle layer. TLR2 and CD14 are not fully colocalized with each other, suggesting that these coelomocytes may belong to two distinct families (Fig. 2). Furthermore, mucous glandular cells were tubulin-positive in the epidermis (Fig. 3). By analyzing the “display profile” function of the confocal microscope, we confirmed the fluorescence peaks of colocalization between tested antibodies.

Quantitative analysis revealed a similar number of positive coelomocytes for the individual antibodies tested, as shown in Table 3.

**Table 3:** Quantitative analysis results (mean values ± standard deviations; *n* = 3)

	**No. of coelomocytes**^**a**^
TLR2 +	745.73 ± 121.86**
CD14 +	517.92 ± 88.43*
α-Tubulin +	698.65 ± 103.81*
TLR2 + CD14	503.27 ± 79.91**
TLR2 + α-Tubulin	658.15 ± 97.17*

^**^p ≤ 0.01

^*^p ≤ 0.05

^a^One-way ANOVA and Student’s t-test were used to compare the means

## Discussion

Host responses against invading pathogens are physiological mechanisms belonging to all living organisms. Since the appearance of the first eukaryotic cells, several defense processes have evolved to guarantee cell integrity, homeostasis, and host survival [25]. Invertebrates have developed a range of defense responses that effectively recognize and remove foreign materials, microbes, or parasites. While chordates have adaptive immunity, lophotrochozoans as earthworms rely primarily on innate immune cells, such as phagocytes (amoebocytes, coelomocytes), that involve phylogenetically conserved PRRs to mediate the immune response [41]. Scavenger receptors, TLRs, and Nod-like receptors (NLRs) are prominent representatives in invertebrates. Following receptor-ligand binding, signal transduction initiates a complex cascade of cellular reactions, leading to the production of one or more effector molecules involved in the immune response [61]. The coelomic cytolytic protein (CCF), the lipopolysaccharide-binding protein (EaLBI/BPI), and the Toll-like EaTLR receptor are the three types of PRRs that have been identified in earthworms so far [65, 66]. CCF has been identified and characterized as an immune molecule of the coelomic fluid and coelomocytes of *Eisenia foetida* (Savigny, 1826). CCF, binding to the microbial antigens, can trigger the prophenoloxidase cascade, an important invertebrate immune mechanism that also exerts opsonizing properties, thus promoting phagocytosis [18, 35].

TLRs are evolutionarily conserved membrane recognition receptors [9, 15] that recognize foreign antigens using PAMPs [2] and contribute to the modulation of the immune response [8, 9, 11, 36]. Bodó et al. [24] demonstrated the presence of TLR on coelomocytes of an annelid oligochaete *Eisenia andrei* (Bouché, 1972) [24]. Skanta et al. (2013) isolated earthworm TLR (EaTLR) from *E. andrei*. The high intraspecies diversity of this receptor indicates to the presence of several TLR genes within the *E. andrei* genome. A TLR from the annelid polychaete *Capitella teleta* (Blake, Grassle, and Eckelbarger, 2009), EaTLR, and TLRs of mollusks and echinoderms share a lot of similarities, according to phylogenetic research [66]. Prochazkova et al. highlighted EaTLR and multiple cysteine cluster (mcc) EaTLR expressed throughout the body tissues of earthworms. The first one is particularly abundant in the digestive system, while the second may act in the embryonic development and the response against parasites [61]. Furthermore, its presence in coelomocytes can be overexpressed due to bacterial accumulation [66]. Coelomocytes of *Lumbricus terrestris* were able to recognize monocystis in vitro as non-self, confirming their capacity of immune recognition [62]. Transcriptomics studies confirm the presence of 18 TLRs in polychaetae annelid *Arenicola marina* (Linnaeus, 1758) [67].

We have previously demonstrated the presence of TLR2 in several vertebrates [4, 11, 12, 16, 46, 54] and urochordates [14, 45].

Following these data, our study characterized immunohistochemically TLR2 in coelomocytes of *Lumbricus terrestris* for the first time. We colocalized TLR2/CD14 and TLR2/α-Tubulin, confirming two cell types of coelomocytes, as reported by Engelmann [33]. Not all coelomocytes were colocalized for the antibodies tested, suggesting a probable functional and structural diversification of these cells. Although there is no experimental evidence, the presence of TLR2/CD14 colocalized coelomocytes may suggest that these cells could be amoebocytes involved in phagocytosis, while coelomocytes expressing only TLR may be eleocytes cells involved in the immune response but not performing phagocytic functions [1, 37, 38],S. J. [49, 50], p.,[57, 71]. Despite Bodó et al. suggest the absence of TLR in *E. andrei* eleocytes using qPCR [23], TLR2-positive eleocytes are detected in *L. terrestris*, suggesting a difference between earthworm species, as already hypothesized on a nuclear genomic base [64].

A study by Eguielor et al. [31] in leech *Glossiphonia complanata* (Linnaeus, 1758) identified morphologically and histochemically three main cell types (macrophage-like cells, NK-like cells, granulocytes type I and II) using different anti-human markers in mouse specific for macrophages CD14 and CD61 [44], and NK cells CD56 and CD57 [43]. This cross-reactivity to antibodies generated against mammalian CD antigens agrees with literature data on phylogenetic correlations between annelids and vertebrates [21, 30] and could be an example of the evolutive conservation of these immune molecules.

Mouse anti-human anti-CD24, anti-tumor necrosis factor (TNF)-α, anti-CD45RA, and anti-CD45RO antibodies have been localized in the coelomocyte surfaces of the earthworm *Eisenia foetida* [30, 34], suggesting that these molecules are highly conserved during phylogenesis. These studies are consistent with previous research demonstrating that coelomocyte surface antigens are cross-reactive with antibodies against CD14, CD11b, and CD11c produced in mammalian *Themiste petricola* (Amor, 1964), characterizing phagocytes positive to anti-CD14, CD11b and CD11c antibodies [21]. In accordance with these data, our results showed phagocytic cells immunoreactive to CD14 in *Lumbricus terrestris*.

Furthermore, we demonstrated the immunopositivity of coelomocytes to α-Tubulin, colocalized with TLR2, showing a link between cytoskeleton, microtubules, and innate immune response mediated by TLR2. Colocalization of TLR2 and α-Tubulin confirms the involvement of tubulin in the transport of TLRs on the membrane surface. A study by Heli Uronen-Hansson et al. [70] exhibited extensive tubulovesicular expression of TLR2 and TLR4 on internal and external monocytes as well as in dendritic cells. Since these receptors are colocalized with α-Tubulin and linked directly to the Golgi complex, it is possible that the microtubules serve as a conduit for the transfer of TLR vesicles [70]. Tubulin is also present in the epidermis, helping to maintain its integrity [27], and intervening in wound healing [73], thus assisting the innate immune system, as also reported by studies on Nematoda *Caenorhabditis elegans* (Maupass, 1900) [27, 69].

Colocalization, confirmed by the “display profile” function of the confocal microscope, corroborates the results obtained, highlighting the peak of fluorescence corresponding to the binding of the antibodies to the corresponding antigens.

In conclusion, our study identifies by confocal microscopy coelomocytes immunoreactive to TLR2, CD14, and α-Tubulin for the first time in Oligochaeta annelid *Lumbricus terrestris*. These data not only validate the important evolutionary conservation of these molecules, but also deepen our knowledge of the internal system of defense of invertebrates, particularly the annelids, and may provide useful indications for future studies, improving the study of the complex innate immune mechanisms of upper vertebrates.

## Data Availability

The data presented in this study are available within the article.

## References

[1] Aderem A, Underhill DM (1999). Mechanisms of phagocytosis in macrophages. Annu Rev Immunol.

[2] Akira S, Uematsu S, Takeuchi O (2006). Pathogen Recognition and Innate Immunity. Cell.

[3] Alesci A, Albano M, Savoca S, Mokhtar DM, Fumia A, Aragona M, Lo Cascio P, Hussein MM, Capillo G, Pergolizzi S, Spanò N, Lauriano ER (2022). Confocal Identification of Immune Molecules in Skin Club Cells of Zebrafish (Danio rerio, Hamilton 1882) and Their Possible Role in Immunity. Biology.

[4] Alesci A, Capillo G, Fumia A, Messina E, Albano M, Aragona M, Lo Cascio P, Spanò N, Pergolizzi S, Lauriano ER (2022). Confocal Characterization of Intestinal Dendritic Cells from Myxines to Teleosts. Biology.

[5] Alesci A, Capillo G, Mokhtar DM, Fumia A, D’Angelo R, Lo Cascio P, Albano M, Guerrera MC, Sayed RKA, Spanò N, Pergolizzi S, Lauriano ER (2022). Expression of Antimicrobic Peptide Piscidin1 in Gills Mast Cells of Giant Mudskipper Periophthalmodon schlosseri (Pallas, 1770). Int J Mol Sci.

[6] Alesci A, Cicero N, Fumia A, Petrarca C, Mangifesta R, Nava V, Lo Cascio P, Gangemi S, Di Gioacchino M, Lauriano ER (2022). Histological and Chemical Analysis of Heavy Metals in Kidney and Gills of Boops boops: Melanomacrophages Centers and Rodlet Cells as Environmental Biomarkers. Toxics.

[7] Alesci, A., Fumia, A., Miller, A., Calabrò, C., Santini, A., Cicero, N., & Lo Cascio, P. (2022). Spirulina promotes macrophages aggregation in zebrafish (Danio rerio) liver. Nat Prod Res, 1–7. 10.1080/14786419.2022.2089883. 10.1080/14786419.2022.2089883 35707902

[8] Alesci, A., Gitto, M., Kotańska, M., Lo Cascio, P., Miller, A., Nicosia, N., Fumia, A., & Pergolizzi, S. (2022). Immunogenicity, effectiveness, safety and psychological impact of COVID-19 mRNA vaccines. Human Immunol, S0198885922001586. 10.1016/j.humimm.2022.08.004. 10.1016/j.humimm.2022.08.004 PMC9359511 35963787

[9] Alesci, A., Lauriano, E. R., Aragona, M., Capillo, G., & Pergolizzi, S. (2020). Marking vertebrates langerhans cells, from fish to mammals. Acta Histochemica, 122(7): 151622. 10.1016/j.acthis.2020.151622. 10.1016/j.acthis.2020.151622 PMC7480233 33066843

[10] Alesci A, Lauriano ER, Fumia A, Irrera N, Mastrantonio E, Vaccaro M, Gangemi S, Santini A, Cicero N, Pergolizzi S (2022). Relationship between Immune Cells, Depression, Stress, and Psoriasis: Could the Use of Natural Products Be Helpful?. Molecules.

[11] Alesci, A., Pergolizzi, S., Capillo, G., Lo Cascio, P., & Lauriano, E. R. (2022). Rodlet cells in kidney of goldfish (Carassius auratus, Linnaeus 1758): A light and confocal microscopy study. Acta Histochemica, 124(3):151876. 10.1016/j.acthis.2022.151876. 10.1016/j.acthis.2022.151876 35303512

[12] Alesci, A., Pergolizzi, S., Fumia, A., Calabrò, C., Lo Cascio, P., & Lauriano, E. R. (2022). Mast cells in goldfish ( *Carassius auratus* ) gut: Immunohistochemical characterization. Acta Zoologica. 10.1111/azo.12417.

[13] Alesci A, Pergolizzi S, Fumia A, Miller A, Cernigliaro C, Zaccone M, Salamone V, Mastrantonio E, Gangemi S, Pioggia G, Cicero N (2022). Immune System and Psychological State of Pregnant Women during COVID-19 Pandemic: Are Micronutrients Able to Support Pregnancy?. Nutrients.

[14] Alesci, A., Pergolizzi, S., Lo Cascio, P., Capillo, G., & Lauriano, E. R. (2022). Localization of vasoactive intestinal peptide and toll‐like receptor 2 immunoreactive cells in endostyle of urochordate *Styela plicata* (Lesueur, 1823). Microsc Res Tech, . 10.1002/jemt.24119. 10.1002/jemt.24119 PMC9324221 35394101

[15] Alesci A, Pergolizzi S, Lo Cascio P, Fumia A, Lauriano ER (2021). Neuronal regeneration: Vertebrates comparative overview and new perspectives for neurodegenerative diseases. Acta Zoologica.

[16] Alesci A, Pergolizzi S, Savoca S, Fumia A, Mangano A, Albano M, Messina E, Aragona M, Lo Cascio P, Capillo G, Lauriano ER (2022). Detecting Intestinal Goblet Cells of the Broadgilled Hagfish Eptatretus cirrhatus (Forster, 1801): A Confocal Microscopy Evaluation. Biology.

[17] Alturkistani HA, Tashkandi FM, Mohammedsaleh ZM (2015). Histological Stains: A Literature Review and Case Study. Global J Health Sci.

[18] Beschin A, Bilej M, Brys L, Torreele E, Lucas R, Magez S, De Baetselier P (1999). Convergent evolution of cytokines. Nature.

[19] Bilej M, De Baetselier P, Beschin A (2000). Antimicrobial defense of the earthworm. Folia Microbiol.

[20] Bilej M, Procházková P, Šilerová M, Josková R (2010) Earthworm Immunity. In: Söderhäll K, edotiro. Invertebrate Immunity, Vol. 708. Boston: Springer; p. 66–79). 10.1007/978-1-4419-8059-5_4. 10.1007/978-1-4419-8059-5_4 21528693

[21] Blanco GAC, Escalada AM, Alvarez E, Hajos S (1997). LPS-induced stimulation of phagocytosis in the sipunculan worm Themiste petricola: Possible involvement of human CD14, CD11B and CD11C cross-reactive molecules. Dev Comp Immunol.

[22] Bodó K, Boros Á, Rumpler É, Molnár L, Böröcz K, Németh P, Engelmann P (2019). Identification of novel lumbricin homologues in Eisenia andrei earthworms. Dev Comp Immunol.

[23] Bodó, K., Ernszt, D., Németh, P., & Engelmann, P. (2018). Distinct immune- and defense-related molecular fingerprints in sepatated coelomocyte subsets of Eisenia andrei earthworms. Invertebrate Survival J, 338–345 . 10.25431/1824-307X/ISJ.V15I1.338-345.

[24] Bodó K, Kellermayer Z, László Z, Boros Á, Kokhanyuk B, Németh P, Engelmann P (2021). Injury-Induced Innate Immune Response During Segment Regeneration of the Earthworm, Eisenia andrei. Int J Mol Sci.

[25] Buchmann, K. (2014). Evolution of Innate Immunity: Clues from Invertebrates via Fish to Mammals. Frontiers in Immunology, 5. 10.3389/fimmu.2014.00459. 10.3389/fimmu.2014.00459 PMC4172062 25295041

[26] Cholewa J, Feeney GP, O’Reilly M, StĂźrzenbaum SR, Morgan AJ, P\lytycz, B.  (2006). Autofluorescence in eleocytes of some earthworm species. Folia Histochem Cytobiol.

[27] Chuang, M., Hsiao, T. I., Tong, A., Xu, S., & Chisholm, A. D. (2016). DAPK interacts with Patronin and the microtubule cytoskeleton in epidermal development and wound repair. ELife, 5: e15833. 10.7554/eLife.15833. 10.7554/eLife.15833 PMC5053806 27661253

[28] Cooper EL (1969). Chronic allograft rejection inLumbricus terrestris. J Exp Zool.

[29] Cooper EL, Kauschke E, Cossarizza A (2002). Digging for innate immunity since Darwin and Metchnikoff. BioEssays.

[30] Cossarizza A, Cooper EL, Suzuki MM, Salvioli S, Capri M, Gri G, Quaglino D, Franceschi C (1996). Earthworm Leukocytes That Are Not Phagocytic and Cross-React with Several Human Epitopes Can Kill Human Tumor Cell Lines. Exp Cell Res.

[31] de Eguileor M, Grimaldi A, Tettamanti G, Valvassori R, Cooper EL, Lanzavecchia G (2000). Different types of response to foreign antigens by leech leukocytes. Tissue Cell.

[32] Engelmann P, Cooper EL, Opper B, Németh P (2011) Earthworm Innate Immune System. In: Karaca A, editor. In: Biology of Earthworms, Vol. 24. Berlin: Springer. p. 229–45. 10.1007/978-3-642-14636-7_14.

[33] Engelmann P, Hayashi Y, Bodó K, Ernszt D, Somogyi I, Steib A, Orbán J, Pollák E, Nyitrai M, Németh P, Molnár L (2016). Phenotypic and functional characterization of earthworm coelomocyte subsets: Linking light scatter-based cell typing and imaging of the sorted populations. Dev Comp Immunol.

[34] Engelmann P, Pál J, Berki T, Cooper EL, Németh P (2002). Earthworm leukocytes react with different mammalian antigen-specific monoclonal antibodies. Zoology.

[35] Field SG, Kurtz J, Cooper EL, Michiels NK (2004). Evaluation of an innate immune reaction to parasites in earthworms. J Invertebr Pathol.

[36] Fumia, A., Cicero, N., Gitto, M., Nicosia, N., & Alesci, A. (2021). Role of nutraceuticals on neurodegenerative diseases: Neuroprotective and immunomodulant activity. Nat Prod Res, 1–18. 10.1080/14786419.2021.2020265. 10.1080/14786419.2021.2020265 34963389

[37] Goldman RD (2001). Worms reveal essential functions for intermediate filaments. Proc Natl Acad Sci.

[38] Harrison RE, Grinstein S (2002). Phagocytosis and the microtubule cytoskeleton. Biochem Cell Biol.

[39] Homa J, Klosowska A, Chadzinska M (2021). Arginase Activity in Eisenia andrei Coelomocytes: Function in the Earthworm Innate Response. Int J Mol Sci.

[40] Icardo JM, Colvee E, Lauriano ER, Capillo G, Guerrera MC, Zaccone G (2015). The structure of the gas bladder of the spotted gar, Lepisosteus oculatus: the gas bladder of *Lepisosteus oculatus*. J Morphol.

[41] Janeway CA (1989). Approaching the Asymptote? Evolution and Revolution in Immunology. Cold Spring Harb Symp Quant Biol.

[42] Josková R, Šilerová M, Procházková P, Bilej M (2009). Identification and cloning of an invertebrate-type lysozyme from Eisenia andrei. Dev Comp Immunol.

[43] Jung HR, Kim MJ, Wee Y-M, Kim JY, Choi MY, Choi JY, Kwon H, Jung JH, Cho YM, Go H, Kim S-Y, Ryu Y-M, Kim YJ, Kim YH, Han DJ, Shin S (2019). CD56+CD57+ infiltrates as the most predominant subset of intragraft natural killer cells in renal transplant biopsies with antibody-mediated rejection. Sci Rep.

[44] Kapellos TS, Bonaguro L, Gemünd I, Reusch N, Saglam A, Hinkley ER, Schultze JL (2019). Human Monocyte Subsets and Phenotypes in Major Chronic Inflammatory Diseases. Front Immunol.

[45] Lauriano, E. R., Aragona, M., Alesci, A., Lo Cascio, P., & Pergolizzi, S. (2021). Toll-like receptor 2 and α-Smooth Muscle Actin expressed in the tunica of a urochordate, Styela plicata. Tissue Cell, 71:101584. 10.1016/j.tice.2021.101584 10.1016/j.tice.2021.101584 34224967

[46] Lauriano ER, Silvestri G, Kuciel M, Żuwa\la, K., Zaccone, D., Palombieri, D., Alesci, A., & Pergolizzi, S.  (2014). Immunohistochemical localization of Toll-like receptor 2 in skin Langerhans’ cells of striped dolphin (Stenella coeruleoalba). Tissue Cell.

[47] Lauriano ER, Żuwa\la, K., Kuciel, M., Budzik, K. A., Capillo, G., Alesci, A., Pergolizzi, S., Dugo, G., & Zaccone, G.  (2016). Confocal immunohistochemistry of the dermal glands and evolutionary considerations in the caecilian, Typhlonectes natans (Amphibia: Gymnophiona). Acta Zoologica.

[48] Lee B-T, Shin K-H, Kim J-Y, Kim K-W. Progress in Earthworm Ecotoxicology. In: Kim YJ, Platt U, editors. Advanced Environmental Monitoring. Dordrecht: Springer; 2008. p. 248–58. 10.1007/978-1-4020-6364-0_19

[49] Lee, S. J., Yoon, B. R., Kim, H. Y., Yoo, S.-J., Kang, S. W., & Lee, W.-W. (2021). Activated Platelets Convert CD14+CD16- Into CD14+CD16+ Monocytes With Enhanced FcγR-Mediated Phagocytosis and Skewed M2 Polarization. Front Immunol, 11: 611133. 10.3389/fimmu.2020.611133. 10.3389/fimmu.2020.611133 PMC7817612 33488616

[50] Li S, Lei Y, Lei J, Li H (2021). All-trans retinoic acid promotes macrophage phagocytosis and decreases inflammation via inhibiting CD14/TLR4 in acute lung injury. Mol Med Rep.

[51] Licata, A., Ainis, L., Martella, S., Ricca, M. B., Licata, P., Lauriano, E. R., & Zaccone, G. (2002). Immunohistochemical localization of nNOS in the skin and nerve fibers of the earthworm Lumbricus terrestris L. (Annelida Oligochaeta). Acta Histochemica, 104(3): 289–295. 10.1078/0065-1281-00650. 10.1078/0065-1281-00650 12389744

[52] Lowe CN, Butt KR, Cheynier KY-M (2016). Assessment of avoidance behaviour by earthworms (Lumbricus rubellus and Octolasion cyaneum) in linear pollution gradients. Ecotoxicol Environ Saf.

[53] Macova S, Harustiakova D, Kolarova J, Machova J, Zlabek V, Vykusova B, Randak T, Velisek J, Poleszczuk G, Hajslova J, Pulkrabova J, Svobodova Z (2009). Leeches as Sensor-bioindicators of River Contamination by PCBs. Sensors.

[54] Marino A, Pergolizzi S, Lauriano ER, Santoro G, Spataro F, Cimino F, Speciale A, Nostro A, Bisignano G (2015). TLR2 activation in corneal stromal cells by *Staphylococcus aureus* -induced keratitis. APMIS.

[55] Misirlioğlu İM, Tsekova R, Stojanović M (2016). On the presence of Lumbricus terrestris Linnaeus 1758 (Oligochaeta, Lumbricidae)on the Balkan Peninsula: Some aspects of ecology and distribution. TURKISH JOURNAL OF ZOOLOGY.

[56] Müller V, de Boer RJ, Bonhoeffer S, Szathmáry E (2018). An evolutionary perspective on the systems of adaptive immunity: Evolution of adaptive immunity. Biol Rev.

[57] Mylvaganam S, Freeman SA, Grinstein S (2021). The cytoskeleton in phagocytosis and macropinocytosis. Curr Biol.

[58] Pergolizzi S, Alesci A, Centofanti A, Aragona M, Pallio S, Magaudda L, Cutroneo G, Lauriano ER (2022). Role of Serotonin in the Maintenance of Inflammatory State in Crohn’s Disease. Biomedicines.

[59] Porchet-Henner E, Vernet G (1992). Cellular immunity in an annelid (Nereis diversicolor, Polychaeta): Production of melanin by a subpopulation of granulocytes. Cell Tissue Res.

[60] Prochazkova, P., Roubalova, R., Dvorak, J., Navarro Pacheco, N. I., & Bilej, M. (2020). Pattern recognition receptors in annelids. Dev Comparat Immunol, 102:103493. 10.1016/j.dci.2019.103493 10.1016/j.dci.2019.103493 31499098

[61] Prochazkova P, Roubalova R, Skanta F, Dvorak J, Pacheco NIN, Kolarik M, Bilej M (2019). Developmental and Immune Role of a Novel Multiple Cysteine Cluster TLR From Eisenia andrei Earthworms. Front Immunol.

[62] Reinhart M, Dollahon N (2003). Responses of coelomocytes from Lumbricus terrestris to native and non-native eukaryotic parasites. Pedobiologia.

[63] Salzet M (2002). Antimicrobial peptides are signaling molecules. Trends Immunol.

[64] Shekhovtsov SV, Ershov NI, Vasiliev GV, Peltek SE (2019). Transcriptomic analysis confirms differences among nuclear genomes of cryptic earthworm lineages living in sympatry. BMC Evol Biol.

[65] Škanta F, Procházková P, Roubalová R, Dvořák J, Bilej M (2016). LBP/BPI homologue in Eisenia andrei earthworms. Dev Comp Immunol.

[66] Škanta F, Roubalová R, Dvořák J, Procházková P, Bilej M (2013). Molecular cloning and expression of TLR in the Eisenia andrei earthworm. Dev Comp Immunol.

[67] Stanovova, M. V., Gazizova, G. R., & Gorbushin, A. M. (2022). Transcriptomic profiling of immune‐associated molecules in the coelomocytes of lugworm *Arenicola marina* (Linnaeus, 1758). J Exp Zool B Mol Dev Evol. 10.1002/jez.b.23135. 10.1002/jez.b.23135 35438249

[68] Swiderska B, Kedracka-Krok S, Panz T, Morgan AJ, Falniowski A, Grzmil P, Plytycz B (2017). Lysenin family proteins in earthworm coelomocytes – Comparative approach. Dev Comp Immunol.

[69] Taffoni, C., Omi, S., Huber, C., Mailfert, S., Fallet, M., Rupprecht, J.-F., Ewbank, J. J., & Pujol, N. (2020). Microtubule plus-end dynamics link wound repair to the innate immune response. ELife, 9:e45047. 10.7554/eLife.45047. 10.7554/eLife.45047 PMC7043892 31995031

[70] Uronen-Hansson H, Allen J, Osman M, Squires G, Klein N, Callard RE (2004). Toll-like receptor 2 (TLR2) and TLR4 are present inside human dendritic cells, associated with microtubules and the Golgi apparatus but are not detectable on the cell surface: Integrity of microtubules is required for interleukin-12 production in response to internalized bacteria. Immunology.

[71] Velle, K. B., & Fritz-Laylin, L. K. (2020). Conserved actin machinery drives microtubule-independent motility and phagocytosis in Naegleria. J Cell Biol, 219(11): e202007158. 10.1083/jcb.202007158. 10.1083/jcb.202007158 PMC7594500 32960946

[72] Wang W, Zhang J, Wu J, Yu R, Zhang Y, Sun L, Gao Y (2021). Acute Toxicity and Ecotoxicological Risk Assessment of Three Volatile Pesticide Additives on the Earthworm—Eisenia fetida. Int J Environ Res Public Health.

[73] Xu, S., & Chisholm, A. D. (2011). A Gαq-Ca2+ Signaling Pathway Promotes Actin-Mediated Epidermal Wound Closure in C. elegans. Curr Biol, 21(23): 1960–1967. 10.1016/j.cub.2011.10.050. 10.1016/j.cub.2011.10.050 PMC3237753 22100061

[74] Zaccone D, Icardo JM, Kuciel M, Alesci A, Pergolizzi S, Satora L, Lauriano ER, Zaccone G (2017). Polymorphous granular cells in the lung of the primitive fish, the bichir P olypterus senegalus. Acta Zoologica.

[75] Zaccone G, Alesci A, Mokhtar DM, Aragona M, Guerrera MC, Capillo G, Albano M, de Oliveira Fernandes J, Kiron V, Sayed RKA, Hussein MM, Lo Cascio P, Kuciel M, Zuwala K, Germanà A, Icardo JM, Lauriano ER (2023). Localization of Acetylcholine, Alpha 7-NAChR and the Antimicrobial Peptide Piscidin 1 in the Macrophages of Fish Gut: Evidence for a Cholinergic System, Diverse Macrophage Populations and Polarization of Immune Responses. Fishes.

